# Artificial Intelligence for Objective Cosmetic Analysis: From Automated Wrinkle Analysis to Precision Facial Phenotyping

**DOI:** 10.1111/jocd.71153

**Published:** 2026-08-19

**Authors:** İstem Şanal Çamur, Eren Çamur

**Affiliations:** ^1^ Department of Dermatology and Venerology Ankara Bilkent City Hospital Ankara Turkey; ^2^ Department of Radiology Ankara 29 Mayıs State Hospital Ankara Turkey

Dear Editor,

We read with great interest the study by Vignoli et al. evaluating the Artificial Intelligence Wrinkle Assessment (AURA‐W) system for objective quantification of facial wrinkle severity after botulinum toxin treatment [1]. The authors should be congratulated for addressing one of the longstanding challenges in aesthetic dermatology: replacing subjective clinical wrinkle grading with reproducible, automated image analysis. By combining standardized three‐dimensional facial photography with an artificial intelligence (AI) system combining automated image segmentation and deep learning–based image analysis, the study demonstrates that AI can objectively quantify treatment‐induced wrinkle changes with high correlation to physician assessments while minimizing observer variability. This study represents an important step toward standardized outcome assessment in cosmetic dermatology.

From a clinical perspective, the proposed framework demonstrates how modern AI can objectively analyze facial images and quantify wrinkle characteristics while reducing observer variability. As AI technology continues to evolve, future systems may achieve greater robustness across different skin phototypes, ethnicities, imaging devices, and clinical environments by incorporating newer image‐analysis approaches that require less task‐specific training data [2, 3]. Integration of these foundation models may further improve robustness across different ethnicities, skin phototypes, imaging devices, and clinical environments.

Another promising direction is the expansion of wrinkle assessment into comprehensive digital facial phenotyping. Because facial aging is inherently multidimensional, emerging AI systems may integrate wrinkle severity with skin texture, pigmentation, vascular features, facial symmetry, soft‐tissue volume, and dynamic facial movement to generate objective composite biomarkers of aging and treatment response [4]. Such multidimensional assessment could improve both clinical research and routine aesthetic practice by providing more comprehensive and reproducible outcome measures.

Beyond objective assessment, AI may also support personalized aesthetic treatment planning by identifying patient‐specific wrinkle patterns, predicting treatment response, and estimating retreatment intervals or individualized dosing strategies [5]. These applications could facilitate AI‐assisted decision support while preserving physician oversight within precision aesthetic medicine [5].

Finally, future AI systems may also benefit from explainability, confidence estimation, and model calibration to improve clinician trust and facilitate regulatory approval and clinical implementation. As multimodal AI continues to evolve, objective wrinkle assessment may become one component of broader decision‐support systems integrating imaging, patient characteristics, and treatment outcomes [6].

In conclusion, this study extends beyond automated wrinkle quantification by illustrating how objective imaging biomarkers may serve as the foundation for future AI‐assisted facial phenotyping and personalized aesthetic decision support. Continued development of clinically interpretable, robust, and externally validated AI systems will be essential to translate these advances into routine cosmetic dermatology practice.

## Funding

The authors have nothing to report.

## Ethics Statement

No patient information and images are used to eliminate the need for ethics committee approval. Ethical approval is not applicable to this letter.

## Conflicts of Interest

The authors declare no conflicts of interest.

## Data Availability

Data sharing not applicable to this article as no datasets were generated or analyzed during the current study.
